# Effects of polycystic ovary syndrome on liver, heart, muscle, and pancreatic-related diseases

**DOI:** 10.3389/fendo.2026.1776584

**Published:** 2026-04-10

**Authors:** Bingrui Li, Yuan Li, Xianglin Li, Zicheng Yu, Jin Yu, Yang Shao, Tonglu Li, Shuai Sun, Ling Zhou, Ruipin Yao, Wen Cheng, Jing Zhu, Jindong Miao, You Li, Yang Wu, Yin Shi, Jing Zhou, Chaoqin Yu, Changquan Ling

**Affiliations:** 1 Basic Medicine School, Naval Medical University, Shanghai, China; 2 Gynecology Department of Traditional Chinese Medicine, The First Affiliated Hospital of Naval Medical University, Shanghai, China; 3 Shanghai Seventh People's Hospital Affiliated to Shanghai University of Traditional Chinese Medicine, Shanghai, China; 4 School of Traditional Chinese Medicine, Navy Medical University, Shanghai, China

**Keywords:** cardio vascular disease, insulin resistance, metabolic dysfunction, pancreatic dysfunction, polycystic ovary syndrome, sarcopenia, systemic inflammation

## Abstract

**Background:**

PCOS is far more than an ovarian disorder; it is a systemic metabolic crisis affecting 11–13% of women. This review maps its extra-ovarian reach into the liver, heart, muscle, and pancreas.

**Methods:**

We analyzed how the "pathophysiological quartet"—insulin resistance, hyperandrogenism, inflammation, and oxidative stress—coordinates systemic damage.

**Results:**

PCOS prevalence skyrockets to 28.3% in women with obesity. Key findings include a 51.61% prevalence of MASLD in obese patients and a 2–4 fold increase in cardiovascular and type 2 diabetes risk. These manifestations are rooted in a shared metabolic "soil," creating a vicious cycle of androgen excess and multi-organ dysfunction, including sarcopenia and β-cell exhaustion.

**Conclusion:**

Fragmented care is insufficient. The systemic nature of PCOS demands a paradigm shift toward integrated, multidisciplinary management, treating the patient as a metabolic whole.

## 1 Introduction

### 1.1 Overview of PCOS

Polycystic ovary syndrome (PCOS) is a complex endocrine disorder affecting 11–13% of women of reproductive age worldwide, imposing significant health and economic burdens (1). It is characterized by three cardinal features: hyperandrogenism, ovulatory dysfunction, and polycystic ovarian morphology. The pathogenesis of PCOS is multifactorial, involving genetic susceptibility, environmental influences, IR, and dysregulation of the hypothalamic–pituitary–ovarian (HPO) axis. Recent studies have further implicated chronic low-grade inflammation, gut microbiota dysbiosis, and neuroendocrine imbalances in the disease process (2–4).

Current management primarily targets symptom control through lifestyle modifications, oral contraceptives for menstrual regulation, insulin sensitizers (e.g., metformin), and ovulation-inducing agents (5). However, given that PCOS affects multiple physiological systems beyond reproduction, a more comprehensive and integrative therapeutic approach is warranted.

### 1.2 Historical evolution of PCOS diagnostics

PCOS is diagnosed primarily according to the 2003 Rotterdam Criteria (6), which require the presence of at least two of the following three features after exclusion of other etiologies: ① ovulatory dysfunction (e.g., oligomenorrhea or amenorrhea); ② clinical or biochemical hyperandrogenism (e.g., hirsutism, acne, or elevated serum androgens); and ③ polycystic ovarian morphology on ultrasonography (≥12 follicles per ovary and/or increased ovarian volume).

These criteria first delineated the multiple clinical phenotypes of PCOS. Subsequent international guidelines have built upon this framework, placing particular emphasis on comprehensive differential diagnosis and metabolic risk assessment (e.g., screening for diabetes and dyslipidemia) in clinical management.

### 1.3 Systemic manifestations beyond reproductive dysfunction

This review focuses on the hepatic, cardiovascular, muscular, and pancreatic complications of PCOS based on several critical considerations. First, these organs represent the primary insulin-sensitive tissues and are therefore most vulnerable to PCOS-related metabolic dysfunction. Epidemiologically, up to 51.61% of obese PCOS patients develop hepatic steatosis (7), cardiovascular disease risk is elevated 2–4 fold (8–10), and type 2 diabetes incidence is similar elevated (11). Second, these organs share common pathophysiological mechanisms with PCOS, including IR, chronic low-grade inflammation, oxidative stress, and lipotoxicity, suggesting interconnected systemic dysfunction rather than isolated complications. Third, the “spillover” hypothesis proposes that metabolic disturbances originating in the ovary systemically affect distal organs through circulating inflammatory mediators, adipokines, and metabolites (12, 13). Finally, recognizing multi-organ involvement has direct clinical implications, supporting the need for multidisciplinary management strategies that extend beyond reproductive health to address cardiovascular, metabolic, and musculoskeletal risks. This integrated perspective may guide the development of therapeutic interventions targeting shared pathophysiological pathways.

At its core, PCOS is a systemic metabolic disease driven by a vicious cycle of hyperandrogenemia and IR (14). Large-scale epidemiological studies have confirmed that patients with PCOS patients face a significantly elevated risk of type 2 diabetes, metabolic dysfunction–associated steatotic liver disease (MASLD), and cardiovascular disease (15). Notably, the pattern of organ involvement varies across clinical phenotypes; for example, the hyperandrogenic phenotype is associated with more severe hepatic steatosis and fibrosis, reflecting the inherent heterogeneity of the disease. Mechanistically, androgen excess directly contributes to visceral adipose dysfunction, skeletal muscle IR, and hepatic lipid accumulation, while impaired glucose metabolism and mitochondrial dysfunction in visceral adipose tissue further exacerbate systemic energy imbalances.

The progression and manifestations of PCOS are therefore closely linked to multi-organ dysfunction, particularly involving the liver, cardiovascular system, skeletal muscle, and pancreas (16). These extra-ovarian manifestations contribute significantly to patient morbidity and mortality.

#### 1.3.1 Hepatic complications

PCOS patients frequently present with metabolic dysfunction–associated steatotic liver disease (MASLD), with a prevalence reaching 51.61% among obese individuals. The ultrasound-derived fat fraction (UDFF) correlates positively with fatty liver severity, reflecting increased hepatic lipid accumulation and inflammation (7, 17).

#### 1.3.2 Cardiovascular manifestations

PCOS contributes to cardiovascular injury through chronic low-grade inflammation and sympathetic overactivation, as evidenced by elevated myocardial macrophage infiltration, accelerated atherosclerotic plaque development, and impaired post-infarction myocardial repair—mechanisms that operate independently of conventional metabolic abnormalities (16). Furthermore, the waist-to-height ratio (WHtR) in PCOS patients is significantly associated with cardiovascular risk indicators (blood pressure, IR, dyslipidemia), potentially further increasing cardiovascular disease risk (18).

#### 1.3.3 Pancreatic involvement

PCOS may be associated with a 1.9-fold higher risk of pancreatic cancer, likely driven by metabolic disturbances beyond obesity or diabetes alone (19). Additionally, PCOS patients exhibit markedly higher incidences of type 2 diabetes, β-cell dysfunction, and acute pancreatitis.

#### 1.3.4 Muscular complications

Although specific studies on muscle involvement in PCOS remain limited, PCOS-related IR and chronic inflammation indirectly impair muscle metabolic function, promoting sarcopenia, intramuscular fat infiltration, and decreased muscle strength, which may in turn exacerbate systemic metabolic dysfunction (17, 20).

**Figure 1:**
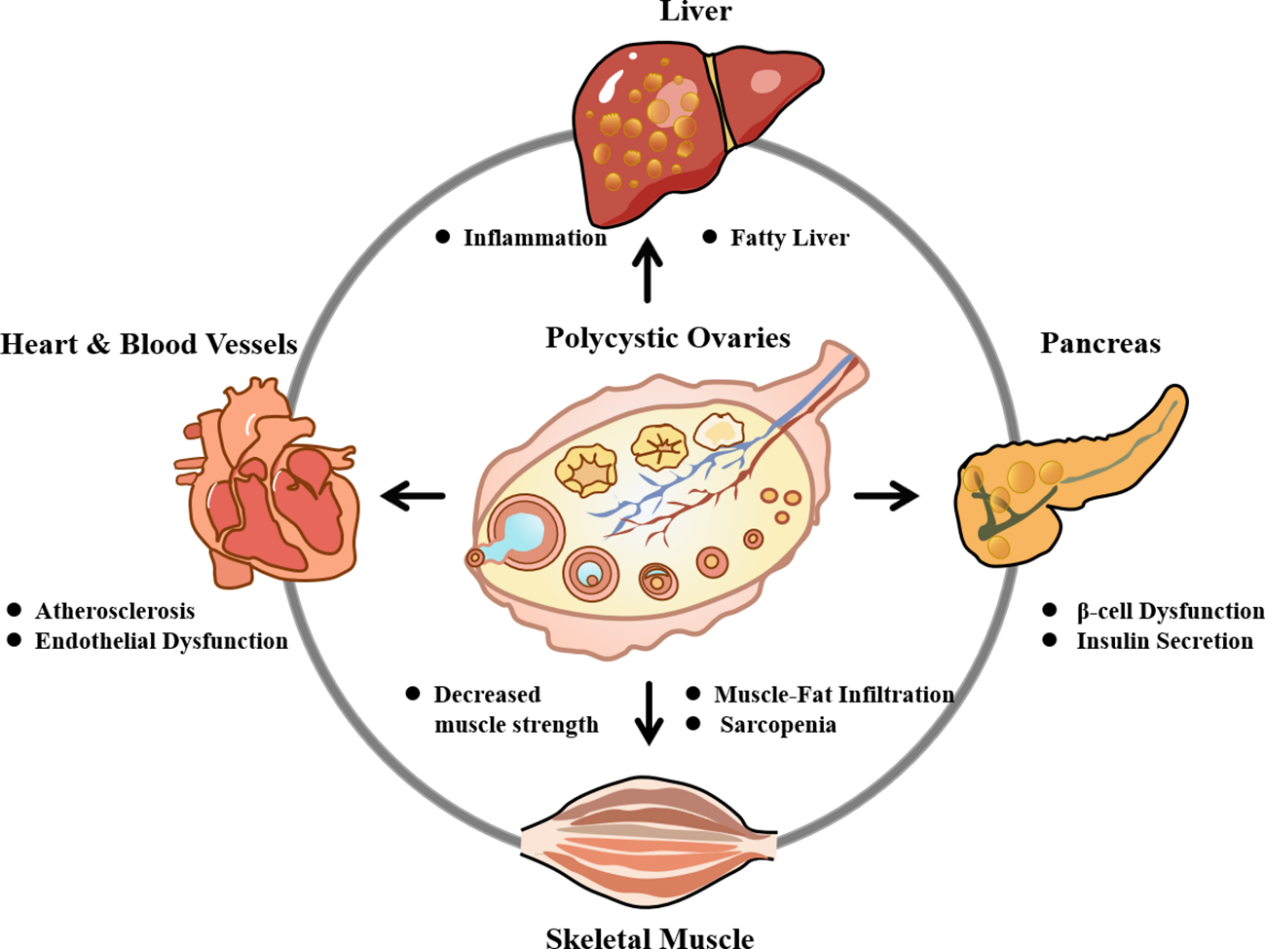
The multi-organ pathophysiology of polycystic ovary syndrome. PCOS is a systemic metabolic disorder originating from ovarian dysfunction but extending its impact to multiple peripheral organs. The central pathology involves polycystic ovaries characterized by hyperandrogenism and chronic inflammation. These core defects establish bidirectional vicious cycles with extra-ovarian tissues: ① Liver: Hyperandrogenism and insulin resistance (IR) drive hepatic steatosis and metabolic dysfunction-associated steatotic liver disease (MASLD). ② Pancreas: Chronic demand for insulin compensation leads to β-cell dysfunction and exhaustion. ③ Skeletal Muscle: Defective insulin signaling contributes to systemic IR and reduced glucose uptake. ④ Heart & Vasculature: Systemic inflammation and endothelial dysfunction accelerate atherosclerosis and increase cardiovascular risk. ⑤ Adipose Tissue: Visceral adiposity secretes pro-inflammatory cytokines (TNF-α IL-6), further exacerbating IR. ⑥ Gut Microbiota: Dysbiosis disrupts the gut-brain-ovary axis, influencing metabolic and hormonal homeostasis. Collective dysfunction in these organs creates a self-perpetuating cycle that aggravates the clinical phenotype of PCOS.

These multi-organ interactions highlight the complexity of PCOS management and emphasize the need for interdisciplinary collaboration to optimize treatment strategies (**Figure 1**).

Beyond the primary physical effects mentioned above, PCOS also increases the risk of kidney disease, primarily through metabolic disorders caused by insulin resistance and chronic low-grade inflammation (21). This leads to glomerular hyperfiltration, tubular damage, and proteinuria, accelerating the progression of chronic kidney disease. Even in PCOS patients without traditional risk factors for kidney injury, mild elevations in glomerular filtration rate and increased urinary albumin excretion are relatively common. Furthermore, PCOS also impacts the respiratory and nervous systems (22, 23), among others, with the underlying mechanisms requiring further investigation.

## 2 Liver complications in PCOS

### 2.1 Pathophysiological mechanisms

Hepatic complications in PCOS primarily include metabolic dysfunction-associated steatotic liver disease (MASLD), abnormal liver function (elevated transaminases), and a progressive risk of liver fibrosis. These complications are closely related to the core pathophysiological mechanisms of PCOS, particularly IR, hyperandrogenism, and chronic low-grade inflammation. Early observational studies have shown that PCOS patients have a significantly higher prevalence of fatty liver and liver function abnormalities compared to healthy individuals, particularly among those who are overweight (24, 25). More recently, non-invasive hepatic steatosis indices have confirmed the high prevalence of MASLD in PCOS populations, with the hepatic steatosis index (HSI) and fatty liver index (FLI) showing strong diagnostic performance (26).

Emerging evidence indicates that hepatic complications in PCOS extend beyond simple metabolic disturbances (hyperinsulinemia and dyslipidemia), encompassing the gut microbiota–bile acid–liver axis. A landmark study published in *Nature Metabolism* (2024) revealed that agmatine, a gut microbiota metabolite primarily derived from *Bacteroides vulgatus*, modulates hepatic metabolism by suppressing glucagon-like peptide-1 (GLP-1) secretion via farnesoid X receptor (FXR) activation, thereby aggravating IR and ovarian dysfunction (27–30).

### 2.2 MASLD in PCOS: the multiple-hit hypothesis

MASLD is the most common hepatic complication associated with PCOS. Both the HSI and FLI demonstrate excellent diagnostic performance (AUC of 0.88 and 0.89, respectively). Patients with MASLD exhibit higher body mass index, insulin resistance, triglyceride levels, and lower testosterone and sex hormone-binding globulin levels (26). Current understanding has evolved beyond the traditional “two-hit hypothesis” to embrace the more comprehensive “multiple-hit hypothesis,” which includes IR, hyperandrogenism, gut microbiota dysbiosis, chronic inflammation, and other metabolic abnormalities (31).

#### 2.2.1 Insulin resistance

IR is a key driver of MASLD development in patients with PCOS. It enhances adipose tissue lipolysis, leading to increased free fatty acid (FFA) influx to the liver, while hyperinsulinemia promotes hepatic *de novo* lipogenesis through activation of the sterol regulatory element-binding protein-1c (SREBP-1c) (32, 33). Notably, the severity of IR in PCOS patients often exceeds that expected from obesity alone, suggesting that PCOS-specific factors independently contribute to MASLD pathogenesis (8).

#### 2.2.2 Hyperandrogenism

Elevated androgen levels in PCOS patients contribute to MASLD through multiple mechanisms. Reduced sex hormone-binding globulin (SHBG) levels increase the bioavailability of free fatty acids, promoting hepatic fat accumulation (9, 10). Androgens also suppress key enzymes involved in fatty acid β-oxidation, such as carnitine palmitoyltransferase 1A (CPT1A), directly impairing lipid metabolism within hepatocytes (31). Furthermore, androgens activate Kupffer cells to secrete pro-inflammatory cytokines (TNF-α, IL-6), aggravating hepatic inflammation and potentially driving progression from simple steatosis to steatohepatitis (34).

#### 2.2.3 Gut microbiota dysbiosis

The gut microbiota composition in PCOS patients differs markedly from that of healthy women, with significant enrichment of *Bacteroides vulgatus*. Its metabolite, agmatine, activates FXR and suppresses GLP-1 secretion, thereby exacerbating IR and ovarian dysfunction, which in turn affects hepatic metabolism. Additionally, certain intestinal microbes produce endogenous ethanol that enters the liver via the portal vein, activating cytochrome P450 2E1 (CYP2E1) and inducing oxidative stress–mediated hepatocellular injury (27).

### 2.3 Abnormal liver function in PCOS

alanine aminotransferase (ALT) and aspartate aminotransferase (AST) levels have been reported in PCOS patients, even when values remain within the normal clinical reference range (27). These subclinical elevations correlate with the degree of IR and other metabolic syndrome components. IR increases hepatic gluconeogenesis, intensifying metabolic stress and promoting hepatocellular damage. It also suppresses hepatic secretion of very low-density lipoproteins (VLDL), resulting in intrahepatic fat accumulation and aggravating steatosis. In parallel, hyperandrogenism decreases SHBG synthesis, inhibits CPT1A-mediated fatty acid β-oxidation, increases FFA availability, and fosters hepatic lipid deposition. This lipid accumulation stimulates pro-inflammatory mediators (TNF-α and IL-6), activates Kupffer cells, and exacerbates hepatocellular injury and fibrosis (34).

The mechanisms underlying hepatic abnormalities in PCOS are complex and multifaceted. Future studies should employ multi-omics approaches to develop personalized interventions capable of delaying progression from subclinical liver dysfunction to fibrosis and cirrhosis.

## 3 Cardiovascular complications in PCOS

Polycystic ovary syndrome not only affects fertility but is also linked to a significantly increased risk of developing various cardiovascular conditions (35–37). The main cardiovascular complications in patients with PCOS include atherosclerosis, myocardial infarction (MI), hypertension, and diastolic cardiac dysfunction. Several pathophysiological mechanisms contribute to the onset of these complications, including IR, hyperandrogenism, chronic inflammation, and metabolic abnormalities. In recent years, studies have further confirmed the role of inflammation-mediated mechanisms in the cardiovascular risk associated with PCOS, independent of traditional metabolic risk factors.

### 3.1 Metabolic-independent inflammatory mechanisms

The traditional paradigm of metabolic dependence has long been regarded as the principal explanation for cardiovascular disease in PCOS. This framework suggests that cardiovascular risk in PCOS is primarily mediated by obesity, endocrine dysregulation, and dyslipidemia (38, 39). The atherogenic plasma index (API), calculated as log (triglycerides/HDL-cholesterol), has emerged as a robust predictor of cardiovascular risk in PCOS patients, correlating strongly with carotid intima-media thickness and inflammatory markers (40). Subsequent evidence revealed that hyperandrogenism substantially increased cardiovascular risk even in women with normal BMI, while more recent research has highlighted the role of inflammatory markers such as C-reactive protein (CRP) and interleukin-6 (IL-6) in mediating the PCOS–cardiovascular disease relationship.

A recent landmark study demonstrated that PCOS mice develop cardiovascular injury driven by chronic inflammation even in the absence of metabolic abnormalities (16). The proposed mechanism involves PCOS-induced expansion of splenic monocytes, enhanced myocardial macrophage infiltration, and aggravated cardiac dysfunction following myocardial infarction. This finding challenges the prevailing notion of metabolic dependence by suggesting that chronic inflammation in PCOS may independently elevate cardiovascular disease risk.

### 3.2 PCOS-associated atherosclerotic risk

Atherosclerosis is characterized by the buildup of lipid-rich plaques (atherosclerotic plaques) within the intima of medium and large arteries, leading to reduced blood flow or vascular occlusion (41). Patients with PCOS face a significantly increased risk of developing atherosclerosis (AS). A retrospective study published in 2025 found that patients with PCOS had a significantly higher atherosclerotic plasma index (AIP) (p < 0.001) and insulin resistance (HOMA-IR score) was strongly and positively correlated with AIP compared with healthy controls (r = 0.294). Multivariate linear regression analysis confirmed that both PCOS diagnosis and HOMA-IR were independent risk factors for elevated AIP. The study also noted that the risk of atherosclerosis in patients increased dramatically when body mass index (BMI) ≥ 35 kg/m². These results suggest that PCOS itself, along with its accompanying insulin resistance and severe obesity, combine to exacerbate the atherosclerotic burden (40).

IR, a hallmark feature of PCOS, stimulates hepatic secretion of VLDL while reducing HDL levels, thereby promoting lipid accumulation within arterial walls. IR also inhibits the PI3K/Akt pathway, decreasing nitric oxide (NO) production, while simultaneously activating the Raf/MAPK pathway to enhance endothelin-1 (ET-1) release, collectively promoting vasoconstriction. Additionally, hyperglycemia-driven accumulation of advanced glycation end products (AGEs) further accelerates vascular inflammation and fibrosis (42, 43).

Elevated androgen levels (e.g., testosterone) reduce SHBG levels, increase FFA concentrations, and promote hepatic lipid accumulation and oxidative stress, thereby contributing to atherogenesis. Androgens also inhibit endothelial nitric oxide synthase (eNOS) activity via the androgen receptor (AR), resulting in endothelial dysfunction and impaired vasodilation. Chronic low-grade inflammation, characterized by elevated TNF-α, IL-6, and CRP levels, further accelerates both the formation and destabilization of atherosclerotic plaques (16). Elucidating these molecular interaction mechanisms may clarify the pathophysiology underlying atherosclerosis in PCOS and identify cytokine biomarkers for assessing elevated atherosclerotic cardiovascular disease (ASCVD) risk.

### 3.3 PCOS-associated risk of diastolic dysfunction

Diastolic dysfunction (DD) in patients with PCOS is a common subclinical cardiovascular abnormality, primarily characterized by impaired left ventricular diastolic filling (reduced E/A ratio), increased myocardial stiffness, and left atrial enlargement. The mechanisms underlying its development involve multiple pathophysiological processes (44, 45).

As a central feature of PCOS, IR significantly impacts cardiac diastolic function. IR is now recognized not merely as a metabolic concern but also as a disruption of cardiac energy metabolism. Moreover, IR in PCOS patients tends to be more pronounced than in typical overweight individuals and appears independent of BMI, which may explain why even lean PCOS patients exhibit diastolic dysfunction.

Hyperandrogenism promotes a substantial influx of fatty acids into cardiomyocytes while impairing mitochondrial β-oxidation, leading to lipid accumulation and the onset of myocardial steatosis, which reduces myocardial compliance. In addition, it inhibits the PI3K/Akt pathway and decreases NO production, resulting in disturbances in coronary microcirculation and subsequently compromising myocardial perfusion (46, 47). Persistent hyperglycemia fosters the accumulation of AGEs, which enhance myocardial collagen cross-linking and contribute to increased myocardial stiffness (48, 49).

The cardiac effects of hyperandrogenemia are sex-specific; female PCOS patients may be more sensitive to androgens and develop diastolic dysfunction earlier in the disease course. Elevated androgen levels inhibit eNOS activity, reducing vasodilation and impairing myocardial relaxation (46). Testosterone also promotes cardiac fibroblast proliferation and increases type I and III collagen deposition, leading to myocardial stiffness and diastolic dysfunction (16). Evidence indicates that these patients may additionally experience myocardial injury driven by chronic inflammation, independent of typical metabolic abnormalities. Future research should prioritize anti-inflammatory and metabolic interventions; for example, combining metformin with GLP-1 receptor agonists (e.g., semaglutide) may protect cardiovascular health by improving insulin sensitivity and reducing inflammation (20).

Exploring targeted inflammatory pathways and personalizing metabolic management approaches are crucial for enhancing cardiovascular health in patients with PCOS.

## 4 Muscular complications in PCOS

Patients with PCOS often present with muscle abnormalities such as sarcopenia, myohypertrophy, intramuscular fat infiltration, reduced muscle strength, and pelvic floor muscle dysfunction. The mechanisms underlying these conditions involve the interaction of multiple factors, including IR, hyperandrogenism, chronic inflammation, and oxidative stress (50, 51).

Skeletal muscle, the largest insulin-sensitive tissue and a key metabolic organ, plays a central role in the pathophysiology of PCOS (52, 53). Under physiological conditions, skeletal muscle accounts for approximately 80% of insulin-mediated glucose uptake and maintains lipid homeostasis through mitochondrial β-oxidation. However, skeletal muscle in PCOS patients undergoes marked metabolic reprogramming, including decreased glucose uptake, impaired fatty acid oxidation, and mitochondrial dysfunction—alterations that are crucial contributors to the development of IR.

Recent studies have increasingly focused on the molecular and clinical implications of muscle abnormalities in PCOS. At the molecular level, hyperandrogenemia, chronic low-grade inflammation, and oxidative stress jointly contribute to the onset and progression of these muscle complications. Clinically, reductions in muscle mass and function correlate with the severity of metabolic disturbances, cardiovascular risk, and reproductive outcomes (54). Importantly, PCOS patients with normal BMI may still present muscle abnormalities, suggesting that these alterations are not solely attributable to obesity.

Furthermore, alterations in the gut microbiota–muscle axis and muscle fiber composition play a crucial role in metabolic dysfunction of the muscles in PCOS (55). Evidence indicates that in PCOS patients, gut microbiota influence muscle tissue through the “intestinal permeability–systemic inflammation–muscle” pathway. In particular, dysbiosis of the gut microbiota enhances the production of endogenous ethanol, which enters the liver and muscle via portal circulation, aggravating oxidative stress and mitochondrial impairment. Additionally, compromised gut barrier function allows endotoxins (lipopolysaccharides, LPS) to enter the bloodstream, inducing systemic low-grade inflammation that further deteriorates muscle health.

### 4.1 The risk of sarcopenia in PCOS

The relationship between PCOS and sarcopenia has recently garnered significant attention. Individuals with PCOS often experience metabolic issues such as IR, androgen excess, and chronic inflammation, which may accelerate muscle loss and increase the risk of sarcopenia, potentially causing premature age-related muscle decline in young women (56, 57).

Skeletal muscle is a vital tissue for insulin-mediated glucose uptake. In PCOS patients, IR impairs GLUT4 transporter function, thereby reducing energy delivery to muscles and contributing to muscle atrophy. Recent studies have shown that skeletal muscle in PCOS accumulates branched-chain amino acids (BCAAs: valine, leucine, and isoleucine), which activate the mTOR pathway, subsequently inhibiting autophagy, worsening IR, and establishing a “metabolic vicious cycle” (58). Moreover, abnormal BCAA metabolism interferes with the TCA cycle, diminishing energy production and promoting muscle fiber atrophy. IR also compromises mitochondrial function, leading to oxidative stress, mitochondrial damage, decreased ATP synthesis, and impaired muscle contractility. In addition, hyperinsulinemia dysregulates the mTORC1 signaling pathway, lowering muscle protein synthesis and activating the ubiquitin-proteasome system, ultimately enhancing protein degradation (59, 60).

It is important to note that the effects of androgens on skeletal muscle are complex and likely dependent on dose, duration, tissue type, and receptor sensitivity. Hyperandrogenemia exerts bidirectional effects on muscle mass, marked by a transient increase in muscle synthesis and a prolonged promotion of muscle catabolism. While androgens (e.g., testosterone) are theoretically recognized for enhancing muscle growth, excessive androgen levels associated with IR in PCOS patients may counteract these anabolic effects. As a result, specific symptoms vary individually from one PCOS patient to another, but the evidence of sarcopenia remains the predominant symptom. When the effects of insulin resistance predominate, it will manifest as sarcopenia. In contrast, when high androgen levels predominate, muscle hypertrophy and rigidity are manifested. Moreover, altered androgen receptor (AR) expression in the skeletal muscle of PCOS patients may contribute to impaired anabolic signaling (61).

### 4.2 Risk of muscle-fat infiltration in PCOS

Muscle fat infiltration (i.e., intramuscular lipid accumulation) in patients with PCOS represents a complex pathological condition involving multiple factors, including hormonal dysregulation, metabolic disturbances, inflammatory responses, and muscle fiber remodeling.

In PCOS patients, IR reduces glucose uptake by skeletal muscles, prompting them to rely more on fatty acids for energy. However, when fatty acid uptake exceeds oxidative capacity, lipids accumulate in muscle cells as triglyceride (TG) structures, leading to the formation of lipid droplets (62). Proteomic studies have shown a marked increase in Perilipin-1 expression—a protein that coats lipid droplets—in the skeletal muscles of PCOS patients, contributing to lipid droplet homeostasis and ectopic fat accumulation (50). Additionally, impaired insulin signaling pathways (e.g., PI3K-AKT) disrupt GLUT4 membrane trafficking, further aggravating glucose metabolism in muscle cells and perpetuating a vicious cycle (63, 64).

Patients with PCOS often develop hyperandrogenemia, in which excess androgens (e.g., testosterone) stimulate androgen receptor (AR) activation in skeletal muscle. This results in an increase in type II (glycolytic) muscle fibers and a decrease in type I (oxidative) fibers. Type I muscle fibers have well-developed mitochondria and are more insulin-sensitive, efficiently oxidizing fats, whereas type II fibers rely primarily on anaerobic metabolism, which tends to promote lipid accumulation (11).

In the presence of inflammation and chronic stress, patients with PCOS exhibit elevated expression of inflammatory mediators such as TNF-α, IL-6, and S100A8 (a calcium-binding protein) in muscle tissue. These factors aggravate local inflammation through activation of the NF-κB pathway and concurrently impair insulin signaling. Moreover, markers of endoplasmic reticulum stress (ERS), including PERK and ATF4, have been detected in the muscle and endometrial tissues of overweight PCOS patients, indicating that activation of the unfolded protein response (UPR) may disrupt normal metabolic function in myocytes. Elevated androgen levels and IR further increase the production of reactive oxygen species (ROS), leading to mitochondrial dysfunction and reduced fatty acid β-oxidation (65, 66).

### 4.3 The risk of decreased muscle strength in PCOS

Patients with PCOS frequently exhibit clinical symptoms of reduced muscle strength, a condition directly attributable to impaired mitochondrial function. This impairment is closely associated with the two primary pathological features of hyperandrogenism and insulin resistance. Mitochondrial dysfunction in PCOS muscle tissue is multifactorial, involving insulin resistance and impaired glucose oxidation. Studies confirm that PCOS patients exhibit reduced mitochondrial DNA content and increased oxidative damage, closely associated with insulin resistance (67). Insulin resistance directly impairs muscle tissue’s ability to uptake and oxidize glucose (68), forcing cells to rely instead on the less efficient fatty acid oxidation pathway for energy production (69).In addition, when excessive free fatty acids overwhelm mitochondrial oxidative capacity, these acids are converted into lipotoxic intermediates such as diacylglycerols (DAGs) and ceramides. These products inhibit insulin signaling pathways, exacerbate insulin resistance, and impair mitochondrial function, thereby creating a vicious cycle of lipotoxicity (70). Furthermore, chronic low-grade inflammation and oxidative stress (elevated ROS) directly damage mitochondrial membranes and respiratory complexes (57–60). Studies have documented up to 50% reduction in respiratory function of skeletal muscle mitochondrial complexes I–IV in metabolic syndrome conditions including PCOS, with mitochondrial swelling and cristae structural disruption forming the structural basis for muscle functional impairment (60). This mitochondrial dysfunction markedly decreases ATP production and contributes to reduced muscle strength and endurance in PCOS patients.

### 4.4 Risk of decreased pelvic floor function in PCOS

Multiple mechanisms contribute to reduced pelvic floor function in patients with PCOS, including hormonal imbalances, metabolic disturbances, inflammatory responses, and biomechanical alterations (71). Excess androgens influence pelvic floor muscle and connective tissue, potentially altering both their structural and functional characteristics. These hormones affect muscle elasticity and contractility, leading to hypertrophy and increased stiffness. Moreover, elevated androgen levels may impair the stability of the pelvic floor’s supportive structures by disrupting collagen metabolism, such as decreasing the ratio of collagen type I to type III (72).

IR is common in patients with PCOS and can result in abnormal glucose metabolism within the pelvic floor muscles, ultimately affecting muscle function and force generation. Moreover, hyperinsulinemia may trigger oxidative stress and fibrosis in pelvic tissues by activating inflammatory pathways such as NF-κB, thereby diminishing muscle contractility (21, 73).

Chronic inflammation in PCOS is characterized by elevated levels of pro-inflammatory factors (including TNF-α, IL-6, and CRP), which reduce the synthesis of type I collagen while enhancing the activity of matrix metalloproteinases (MMPs) (74, 75). This process accelerates collagen degradation and weakens the structural integrity of pelvic floor tissues. At the same time, oxidative stress (increased ROS) and inflammation interact synergistically, leading to lipid peroxidation damage in the pelvic floor muscles and fascia, impairing their elasticity and contractile capacity.

Moreover, elevated androgen levels in PCOS patients activate macrophages and stimulate the release of IL-6 and TNF-α, further aggravating systemic low-grade inflammation. Persistent inflammatory stimulation may cause fibrosis and degradation of pelvic floor support structures. Obese PCOS patients often exhibit visceral fat accumulation, which increases intra-abdominal pressure. In addition, pelvic tilt and an increased angle of lumbar lordosis may occur. Prolonged mechanical load can lead to overstretching of the pelvic floor muscles and ligaments, accelerating the development of pelvic floor disorders such as stress urinary incontinence and pelvic organ prolapse.

## 5 Pancreatic complications of PCOS

Polycystic ovary syndrome (PCOS) is a prevalent hormonal and metabolic disorder. Beyond reproductive abnormalities, its impact on the pancreas—including type 2 diabetes, impaired pancreatic β-cell function, acute pancreatitis, and nonalcoholic fatty pancreatic disease—poses serious challenges to patients’ long-term health (30, 76). Pancreatic pathologies in PCOS involve multiple mechanisms, such as disrupted insulin signaling, oxidative stress, chronic inflammation, and gut microbiota imbalance.

Recent research indicates that pancreatic β-cell dysfunction is a key contributor to the metabolic deterioration observed in PCOS. Moreover, anti-inflammatory, antioxidant, and insulin-sensitizing therapies (e.g., metformin and GLP-1 receptor agonists) show potential for improving pancreatic function (77–80).

### 5.1 The risk of type 2 diabetes in PCOS

Women with PCOS have a 2- to 4-fold higher risk of developing type 2 diabetes mellitus (T2DM) compared with unaffected women, with approximately 50% developing diabetes before age 40. The oral glucose tolerance test (OGTT) and insulin secretion assessments are essential for early screening (81).

Several animal studies confirm that insulin resistance plays a central role in the pathogenesis of T2DM. Normally, when insulin binds to its receptor, it activates the PI3K/Akt signaling pathway, promoting GLUT4 translocation to the cell membrane to facilitate glucose uptake into cells (82). However, in PCOS, IR impairs this pathway, reducing glucose transport and uptake. Moreover, the combined effects of PCOS suppress the function of AMP-activated protein kinase (AMPK). AMPK typically promotes fatty acid oxidation and inhibits lipid synthesis by phosphorylating acetyl-CoA carboxylase (ACC) and regulating carnitine palmitoyltransferase 1 (CPT1) (83, 84). As a primary regulator of cellular energy homeostasis, AMPK is frequently suppressed in PCOS. Studies confirm reduced AMPK activity in skeletal muscle, liver, and adipose tissue of PCOS patients, leading to: ① decreased hepatic fatty acid β-oxidation and increased lipid accumulation; ② enhanced lipogenesis through release of SREBP-1c inhibition; ③ impaired muscle glucose uptake due to GLUT4 transport disruption; ④ increased hepatic gluconeogenesis. This inhibition impairs its ability to regulate glucolipid metabolism and amplifies inflammatory and oxidative stress damage (85), thereby driving IR toward diabetes at multiple levels. In addition, it diminishes the suppression of hepatic glucose production (HGP), leading to enhanced gluconeogenesis and elevated fasting blood glucose levels (86).

Notably, metformin—a first-line treatment for PCOS—exerts its beneficial effects partly through AMPK activation, improving insulin sensitivity and reducing hepatic steatosis (87). The therapeutic potential of AMPK activators (including metformin, thiazolidinediones, and novel agents) represents a promising strategy for managing metabolic complications in PCOS.

### 5.2 Hyperinsulinemia and β-cell dysfunction

Hyperinsulinemia is a compensatory response to IR in patients with polycystic ovary syndrome (PCOS); however, prolonged hyperinsulinemia can also lead to reduced β-cell function. Moreover, β-cell dysfunction occurs independently of obesity and is characterized by impaired or severely deficient insulin secretion.

In the early stages of IR, β-cells maintain blood glucose levels through increased insulin secretion, which mainly results in elevated fasting insulin levels. Additionally, neuropeptide dysregulation, such as abnormal glucagon-like peptide-1 (GLP-1) secretion, affects β-cell glucose sensitivity (27). In the decompensated phase of IR, the high metabolic stress accompanying excessive insulin secretion leads to excessive accumulation of reactive oxygen species (ROS) in β-cells, resulting in intense oxidative stress. ROS induces apoptosis through multi-level mechanisms: Beyond directly attacking mitochondria, increasing their membrane permeability, and releasing pro-apoptotic factors like cytochrome c (Cyt c) (88), ROS also persistently activates stress kinase pathways such as c-Jun N-terminal kinase (JNK) and p38 mitogen-activated protein kinase (p38 MAPK). Activated JNK phosphorylates and inhibits the activity of the anti-apoptotic protein Bcl-2, while simultaneously promoting the oligomerization of the pro-apoptotic protein Bax on the mitochondrial membrane. This forms pores that further amplify the apoptotic signal (89, 90). Additionally, ROS can disrupt endoplasmic reticulum homeostasis, triggering the apoptotic pathway of the unfolded protein response (UPR) (91), and these signals converge and activate the key apoptosis-executing protein, caspase-3, thus inducing apoptosis.

### 5.3 The risk of acute pancreatitis in PCOS

The elevated risk of acute pancreatitis in PCOS patients is primarily associated with severe hypertriglyceridemia (HTG). When serum triglyceride levels exceed 1000 mg/dL, lipoprotein lipase in pancreatic capillary beds hydrolyzes large quantities of triglycerides, generating free fatty acids (FFAs). These FFAs locally activate the TLR4/NF-κB pathway in pancreatic acinar cells, promoting the release of pro-inflammatory mediators, triggering the opening of the mitochondrial permeability transition pore that leads to cellular apoptosis, and stimulating pancreatic stellate cells, which in turn contribute to fibrosis and microthrombus formation detrimental to acinar cells (92, 93).

Moreover, PCOS patients frequently present with “metabolic hyperlipidemia,” characterized by IR and increased levels of apolipoprotein C-III (Apo C-III). Apo C-III exacerbates hyperlipidemia by inhibiting lipoprotein lipase activity and impairing hepatic clearance of triglyceride-rich lipoproteins (94–96).

### 5.4 Risk of nonalcoholic fatty pancreas disease with PCOS

Studies have shown that PCOS patients have 30–40% higher pancreatic fat content than BMI-matched healthy women, with a positive correlation to the HOMA-IR index (97, 98).

Under conditions of IR, the PI3K/Akt signaling pathway is impaired in PCOS patients, leading to increased secretion of adipose tissue lipases (e.g., hormone-sensitive lipase, HSL) and enhanced release of FFAs, resulting in a substantial influx of FFAs into the bloodstream. Because the pancreas receives abundant blood flow and exhibits active fatty acid metabolism, FFAs tend to accumulate in the pancreatic parenchyma, causing steatosis. Moreover, excessive androgen levels promote visceral fat accumulation, which further exacerbates IR and FFA release in a vicious cycle. Several animal studies have shown that testosterone acts directly on pancreatic islet β-cells by inhibiting the expression of PDX-1, a key transcription factor in pancreatic development, which may contribute to β-cell dedifferentiation and fatty infiltration (66). Under sustained inflammation and oxidative stress, the buildup of fatty acids in the pancreas results in mitochondrial dysfunction, the generation of ROS, activation of NF-κB pathways, and the release of pro-inflammatory cytokines (such as TNF-α and IL-6), ultimately contributing to pancreatic cell damage (99, 100).

## 6 Systemic integration and inter-organ crosstalk: pathophysiological mechanisms of multi-organ dysfunction

**Figure 2:**
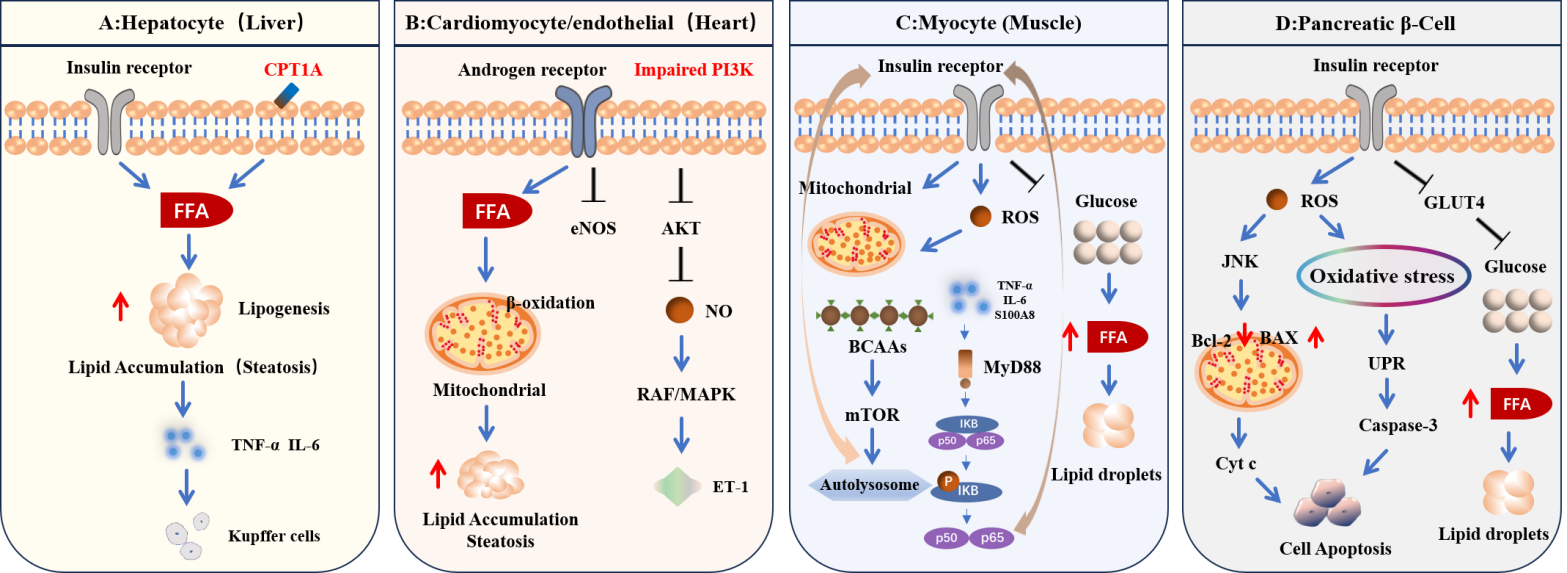
Molecular mechanisms underlying multi-organ complications in PCOS. These effects are closely associated with the fundamental pathophysiological mechanisms of PCOS, particularly IR and hyperandrogenism. The figure illustrates specific molecular pathways by which IR and hyperandrogenism impact target organs including alter actions in SHBG, CPT1A, SREBP-1c, eNOS, PI3K/AKT signaling, NF-κB signaling and other key mediators. **(A)** Hepatocyte: IR enhances adipose lipolysis and increases FFA influx to the liver. Androgens suppress CPT1A, reducing β-oxidation and promoting FFA availability and hepatic fat deposition. TNF-α and IL-6 activate Kupffer cells, worsening hepatocellular injury and fibrosis. **(B)** Cardiomyocyte & Endothelial Cell: Hyperandrogenism drives lipid influx into cardiomyocytes, impairing mitochondrial β-oxidation and causing myocardial steatosis, thereby reducing compliance. IR activates PI3K/Akt (decreasing NO production) while Raf/MAPK activation increases ET-1, leading to vasoconstriction. Androgens suppress eNOS via AR, causing endothelial dysfunction. **(C)** Myocyte: TNF-α, IL-6, and S100A8 activate NF-κB, exacerbating local inflammation and impairing insulin signaling. BCAAs accumulate and activate mTOR, inhibiting autophagy and worsening IR. IR impairs mitochondrial function and forces reliance on fatty acids; excess FFA uptake leads to intramyocellular lipid accumulation. **(D)** Pancreatic β-Cell: IR reduces GLUT4 at the cell membrane, shifting energy production to FFAs. Excess FFA intake causes intracellular lipid droplet accumulation, excessive ROS, and oxidative stress. ROS activates JNK, promotes Bax oligomerization on the mitochondrial membrane, triggers Cyt release and caspase-3 activation, and ultimately induces apoptosis. ROS also disrupts ER homeostasis, triggering the UPR.

The connection between polycystic ovary syndrome and its extrahepatic manifestations—including metabolic syndrome, type 2 diabetes, and cardiovascular diseases—represents a complex interplay driven by IR, hormonal disturbances, chronic inflammation, and oxidative stress (**Figure 2**).

### 6.1 Insulin resistance

IR is the core driver of PCOS’s systemic impact, characterized by reduced insulin sensitivity in skeletal muscle, liver, and adipose tissue, forming “selective IR.” This leads to critical inter-organ communication disruptions: (1) Adipose-Liver Axis: IR promotes lipolysis in adipose tissue, releasing excessive FFAs into the liver. This drives hepatic lipid synthesis and inhibits VLDL secretion, leading to hepatic lipid accumulation and promoting MASLD (32, 33). (2) Liver-systemic axis: Steatotic liver secretes inflammatory factors (e.g., CRP) and abnormal lipoproteins, exacerbating inflammation and IR in distal organs (e.g., vasculature, muscle) via the circulation (16). (3) Hyperinsulinemia-Ovarian Axis: Circulating hyperinsulinemia directly stimulates ovarian androgen synthesis while simultaneously suppressing hepatic SHBG production, elevating free androgen levels and creating a vicious cycle (20). Thus, IR represents not merely a metabolic defect but a core communication failure disrupting multi-organ energy homeostasis and hormonal equilibrium (101).

### 6.2 Hyperandrogenism

Hyperandrogenemia extends beyond reproductive endocrinology, acting as a direct “tissue remodeling factor” that causes specific damage to multiple target organs via AR-mediated mechanisms. In the liver, androgens inhibit CPT1A, a key enzyme in fatty acid β-oxidation, while activating Kupffer cells to release TNF-α and IL-6. Together, these processes promote lipid accumulation and inflammation, driving the progression of MASLD (33, 36). In the cardiovascular system, androgens inhibit endothelial eNOS via AR, impairing vasodilation; they also stimulate collagen deposition in cardiac fibroblasts, increasing myocardial stiffness—a key mechanism underpinning diastolic dysfunction (18, 48). In skeletal muscle, androgens promote the conversion of muscle fibers from oxidative (type I) to glycolytic (type II) types, reducing overall insulin sensitivity and lipid oxidation efficiency, thereby creating conditions for intramuscular lipid infiltration (13). In the pancreas, animal studies indicate testosterone may directly impair β-cell function (68).

There is a bidirectional relationship between androgen excess and metabolic disorders. On one hand, androgens can worsen the inflammatory response and aggravate abnormalities in IR and lipid metabolism through multiple pathways. On the other hand, hyperinsulinemia can, in turn, intensify hyperandrogenism via various mechanisms (102, 103). This vicious cycle amplifies metabolic and endocrine disturbances in PCOS patients, leading to progressive deterioration. Collectively, hyperandrogenism constitutes a hormonal signaling network that directly attacks the structure and function of multiple organs.

### 6.3 Chronic inflammation and oxidative stress

Chronic low-grade inflammation and oxidative stress characterize PCOS, persisting even in non-obese patients, and serve as a “common ground” amplifying and transmitting multi-organ damage (104). Adipose tissue (particularly visceral fat) and activated immune cells (such as splenogenic monocytes) constitute major inflammatory sources, continuously releasing cytokines including TNF-α, IL-6, and CRP (18, 25). These inflammatory mediators circulate through the bloodstream, establishing a systemic inflammatory communication network: in the heart, they drive myocardial macrophage infiltration, exacerbating post-ischemic injury independently of metabolic abnormalities (18); in vascular walls, they accelerate atherosclerotic plaque formation and destabilization; in muscles and the pancreas, they locally activate pathways like NF-κB, disrupt insulin signaling, and induce cellular dysfunction (59, 75). Concurrently, mitochondrial dysfunction induced by IR and hyperandrogenism generates ROS. ROS not only directly damage cells but also form positive feedback loops with inflammatory pathways, collectively impairing cellular energy metabolism and integrity across organs.

### 6.4 Gut microbiota

Gut dysbiosis constitutes an emerging, bottom-up axis of inter-organ communication. Altered abundance of specific gut bacteria (e.g., Bacteroides vulgatus) in PCOS patients enables their metabolites to act as key long-range messengers regulating organ function (27, 105). First, direct metabolic effects: Agmatine activates hepatic FXR to suppress GLP-1 secretion, exacerbating systemic IR and ovarian dysfunction (29); Changes in GDCA/TUDCA may affect intestinal immunity by suppressing IL-22 production and disrupting barrier function (106). Second, endotoxin translocation: Dysbiosis damages the intestinal barrier, allowing endotoxins like LPS to enter the circulation and trigger systemic low-grade inflammation. This inflammatory signal can spread to multiple organs including the liver and muscles (27). Additionally, endogenous ethanol produced by certain microbiota can directly reach the liver and muscles via the portal vein, inducing oxidative stress (29). Thus, the gut microbiota, through its metabolome, serves as a crucial upstream hub regulating hepatic metabolism, systemic inflammation, and insulin sensitivity.

## 7 Innovative and comprehensive approaches for managing PCOS and associated extrahepatic complications

Polycystic ovary syndrome is a common endocrine metabolic disorder that necessitates a comprehensive multidisciplinary approach for treatment. This strategy includes pharmacological therapy, dietary modifications, and lifestyle interventions to improve metabolic abnormalities, restore ovulatory function, and reduce the risk of long-term complications.

Medication should be individualized according to the patient’s specific symptoms. In obesity and dyslipidemia, lipid-lowering medication (such as statins) should be initiated when lifestyle interventions prove ineffective. Statins not only effectively lower total cholesterol and LDL-cholesterol while improving lipid profiles, but their anti-inflammatory and plaque-stabilizing effects also help reduce the long-term risk of ASCVD, providing direct protection to the cardiovascular system (107). For IR, metformin as a first-line insulin sensitizer not only improves glucose uptake and utilization in peripheral tissues and reduces hyperinsulinemia, but also indirectly lowers ovarian-derived androgen levels (20). Its metabolic benefits may extend to the liver (reducing hepatic glucose output, improving non-alcoholic fatty liver disease risk) and pancreas (protecting β-cell function). Building on this, combination therapy with drugs like inositol can further enhance oocyte quality and insulin signaling, synergistically boosting insulin sensitivity (108). To regulate menstrual cycles, combined oral contraceptives (e.g., formulations with 20–30 mcg ethinyl estradiol) or cyclic progestins (e.g., dydrogesterone) can induce regular endometrial shedding, effectively preventing endometrial hyperplasia. Additionally, COCs directly alleviate symptoms of hyperandrogenism such as hirsutism and acne by suppressing gonadotropin secretion and increasing sex hormone-binding globulin (SHBG) levels (108, 109). In obese PCOS patients with IR, GLP-1 receptor agonists are effective drugs for weight loss and improvement of IR. Their benefits extend broadly across multiple organs: they promote insulin secretion and protect β-cells in the pancreas; may offer cardiovascular benefits independent of glucose control (such as improving heart failure outcomes and reducing major cardiovascular event risk); aid in reducing hepatic steatosis and inflammation in the liver; and weight loss itself indirectly improves insulin sensitivity in muscle tissue (110). Spironolactone is a commonly used option for treating hyperandrogenism. It competitively inhibits androgen receptors in the sebaceous follicular units (111). However, as a potassium-sparing diuretic, it carries a risk of causing hyperkalemia. Therefore, it must be used under medical supervision with regular monitoring of serum potassium levels and renal function.

Dietary adjustments play a central role in the management of PCOS. Modern dietary patterns characterized by high-glycemic index (GI) carbohydrates and high fat intake jointly drive the core pathophysiological changes of PCOS through multiple pathways. On one hand, such diets directly promote caloric excess and abdominal obesity (particularly visceral fat accumulation). On the other hand, they trigger dramatic fluctuations in postprandial blood glucose and insulin levels while exacerbating chronic low-grade inflammation. Visceral fat is not merely a passive energy storage organ but an active endocrine organ. It secretes abnormal adipokines, such as reduced insulin-sensitizing adiponectin and elevated pro-inflammatory factors (e.g., TNF-α, IL-6), directly exacerbating systemic insulin resistance. Insulin resistance and hyperinsulinemia further stimulate excessive androgen secretion by the ovaries and adrenal glands while suppressing hepatic synthesis of sex hormone-binding globulin. This leads to elevated free androgen levels, creating a vicious cycle of “insulin resistance -hyperandrogenism -abdominal obesity” (12, 112).

Therefore, targeted dietary interventions are key to breaking this cycle. Reducing daily calorie intake by 500–750 kcal aids in weight loss and visceral fat reduction. Dietary structure should strictly limit refined carbohydrates with high GI and saturated fats to stabilize blood sugar and alleviate insulin resistance. Simultaneously, actively increasing foods rich in omega-3 polyunsaturated fatty acids is recommended, as their anti-inflammatory properties help improve insulin sensitivity. Furthermore, vitamin D supplementation plays a positive role in improving insulin resistance, regulating hormonal balance, and supporting follicular development in PCOS patients. Collectively, dietary patterns characterized by low GI, high fiber, quality fats, and adequate micronutrients—such as the Mediterranean diet—have been demonstrated to effectively improve metabolic parameters, hormone levels, and reproductive function in PCOS patients (113–115).

Exercise is also a key component of PCOS management. It is recommended to engage in at least 150 minutes of moderate aerobic activity (such as brisk walking or swimming) per week, complemented by strength training 2–3 times weekly to improve insulin sensitivity and decrease body fat. A weight loss of 5–10% can markedly enhance hormone balance and ovulatory function (116). Furthermore, optimizing sleep (7–8 hours per night) and managing stress (e.g., through meditation or yoga) can help reduce chronic inflammation and IR. Cognitive-behavioral interventions can further support patients in developing sustainable healthy habits and improving treatment adherence (117).

## 8 Conclusions

Managing PCOS requires a comprehensive, individualized approach integrating lifestyle modifications, pharmacological interventions, and emerging therapies to address both ovarian and systemic manifestations. Central to this strategy is the recognition that PCOS is a multifaceted disorder with significant implications for cardiovascular health, skeletal muscle function, and pancreatic integrity. The interconnected nature of PCOS and its extra-ovarian effects underscores the need for a multidisciplinary care model that prioritizes early detection, precise risk assessment, and personalized treatment to reduce the progression of associated conditions.

Key lifestyle adjustments, including optimized nutrition, regular physical activity, and effective weight management, form the foundation of PCOS management. These measures help address metabolic disturbances, such as IR and hormonal imbalances. Furthermore, targeting IR, hyperandrogenism, inflammation, and oxidative stress through specific pharmacological therapies is essential for improving long-term outcomes. As research progresses, the development of novel therapies that act on defined molecular pathways offers promise for more precise and effective treatment options, enhancing patient outcomes and decreasing the burden of PCOS-related complications.

Future investigations should focus on identifying novel therapeutic targets, applying precision medicine approaches, and elucidating the long-term impact of PCOS on multi-organ health. Collaborative efforts among hepatologists, endocrinologists, cardiologists, and nutritionists will be indispensable for advancing comprehensive strategies in PCOS management.
